# Approaches and outcomes of adalimumab discontinuation in patients with well-controlled inflammatory arthritis: a systematic search and review

**DOI:** 10.1186/s12969-024-01046-3

**Published:** 2024-12-30

**Authors:** Erin Balay-Dustrude, Jessica Fennell, Kevin Baszis, Y. Ingrid Goh, Daniel B. Horton, Tzielan Lee, Chloe Rotman, Anna Sutton, Marinka Twilt, Olha Halyabar, for the CARRA JIA Inactive Disease Workgroup

**Affiliations:** 1 Seattle Children’s Hospital and Research Center, Seattle, WA USA; 2 Pediatrics, Division of Pediatric Rheumatology, University of Washington, Seattle, WA USA; 3 Pediatric Rheumatology, Connecticut Children’s, Department of Pediatrics, University of Connecticut, Hartford, CT USA; 4 Division of Rheumatology & Immunology, Department of Pediatrics, Washington University School of Medicine, Saint Louis Children’s Hospital, Saint Louis, MO USA; 5 Division of Rheumatology, The Hospital for Sick Children, Toronto, ON Canada; 6 Child Health Evaluative Sciences, SickKids Research Institute, Toronto, ON Canada; 7 Department of Pediatrics, Rutgers Robert Wood Johnson Medical School, New Brunswick, NJ USA; 8 Rutgers Center for Pharmacoepidemiology and Treatment Science, Institute for Health, Health Care Policy and Aging Research, New Brunswick, NJ USA; 9 Department of Biostatistics and Epidemiology, Rutgers School of Public Health, Piscataway, NJ USA; 10 Pediatric Rheumatology, Stanford Medicine Children’s Health, Stanford University School of Medicine, Stanford, CA USA; 11 Medical Library, Boston Children’s Hospital, Boston, MA USA; 12 Department of Epidemiology, University of Washington, Seattle, WA USA; 13 Department of Pediatrics, Alberta Children’s Hospital, Cumming School of Medicine, University of Calgary, Calgary, AB Canada; 14 Division of Immunology, Boston Children’s Hospital, Harvard Medical School, Boston, MA USA

**Keywords:** Adalimumab, Medication discontinuation, Rheumatoid arthritis, Spondyloarthritis, Juvenile idiopathic arthritis

## Abstract

**Objective:**

This systematic search and review aimed to evaluate the available literature on discontinuation of adalimumab and other tumor necrosis factor inhibitors (TNFi) for patients with well-controlled chronic inflammatory arthritides.

**Methods:**

We conducted a publication search on adalimumab discontinuation from 2000–2023 using PubMed, CINAHL, EMBASE, and Cochrane Library. Included studies evaluated adalimumab discontinuation approaches, tapering schemes, and outcomes including successful discontinuation and recapture after flare, in patients with well-controlled disease. Studies included evaluated rheumatoid arthritis, ankylosing spondylitis, psoriatic arthritis, and juvenile idiopathic arthritis (JIA).

**Results:**

Forty-nine studies were included. Studies evaluating adalimumab alone were limited, and many reported TNFi outcomes as a single entity. Studies on rheumatoid arthritis (RA) (32, 8 RCTs) reported flare rates from 33–87%. Flares with medication tapering were slightly lower than with abrupt stop, and successful recapture was generally high (80–100%). Studies on spondyloarthropathy (12, 4 RCTs), focused on tapering, noting lower flare rates in tapering rather than abruptly stopping, and high recapture rates (~ 90%). Studies on JIA (5) were observational and demonstrated modestly lower flare rates with tapering (17–63%) versus abrupt stopping (28–82%). There was notable variability in study design, follow-up duration, specificity for TNFi results, and controlled pediatric studies.

**Conclusion:**

The literature evaluating adalimumab and other TNFi discontinuation, flare rates, and recapture success within the inflammatory arthritis population demonstrated less flare when medications were tapered, over abrupt stop in the RA, spondyloarthropathy, and JIA populations. When medications were restarted after flare, recapture of well-controlled disease was generally high in RA and spondyloarthropathy, and generally favorable in JIA.

**Supplementary Information:**

The online version contains supplementary material available at 10.1186/s12969-024-01046-3.

Biologic disease-modifying antirheumatic drugs (bDMARDs) are widely used for the treatment of inflammatory arthritis. Use of bDMARDs in combination with conventional synthetic DMARDs (csDMARDS) has improved the likelihood of achieving low disease activity (LDA) or remission [1–3], prompting consideration for tapering medications.

Adalimumab, a tumor necrosis factor inhibitor (TNFi), received approval from the U.S. Food and Drug Administration for use in rheumatoid arthritis (RA) in 2002, ankylosing spondylitis in 2006, and juvenile idiopathic arthritis (JIA) in 2008. Many patients treated with adalimumab achieve inactive disease, with reports ranging from 46–77% [4–6]. Once the disease is well controlled, there is a lack of consensus surrounding discontinuation strategies.

The American College of Rheumatology (ACR) 2021 and the European Alliance of Associations for Rheumatology (EULAR) 2022 RA guidelines [7, 8] conditionally recommend gradual discontinuation of DMARDs for patients in LDA or remission for at least six months. The 2023 EULAR psoriatic arthritis guidelines [9] recommend cautious consideration for tapering all DMARDs following sustained remission. The 2019 ACR guidelines for ankylosing spondylitis [10] conditionally recommend against bDMARD discontinuation in patients with stable disease. There is no consensus for bDMARD discontinuation for the JIA population [11–13].

Despite the unknowns, patients and caregivers often desire to withdraw bDMARDs [12, 14]. These aspirations are weighed against risks of disease flares and reattainment of disease control [13, 15]. Better information about discontinuation approaches and outcomes would inform these decisions.

We aimed to perform a review of the available literature for adalimumab discontinuation in JIA. However, given the paucity of available literature in this population, we chose to include individuals with RA and spondyloarthropathy, with the goal of describing tapering strategies, flare rates, and recapture strategies after discontinuation.

In this review, the term taper refers to medication administration change by interval spacing or dose adjustments. Abrupt stop refers to medication discontinuation without dose or interval adjustment. The term discontinuation is used to describe the general intent of de-prescribing medication.

## Methods

Eligible studies reported adalimumab discontinuation in the treatment of chronic inflammatory arthritis and were published January 2000-March 2023.

The systematic search intended to report adalimumab data exclusively. However, it was noted that many reported adalimumab use in their methods but combined all TNFi as a single outcome. Given this limitation, we report available literature that included adalimumab-specific data in their methods but allowed for report of TNFi in their results. Included studies presented data on drug discontinuation in chronic inflammatory arthritis secondary to well-controlled disease, defined as LDA, inactive disease, or remission. Editorials, reviews, abstracts, case reports, drug efficacy trials, non-English publications, and preclinical studies were excluded.

The literature search was conducted in September 2021 and updated in March 2023 using PubMed, CINAHL, EMBASE, and the Cochrane Library. The framework was guided by Arksey et al*.* [16]. Keywords and indexing terms for the search were developed by a medical librarian (CR) and clinicians (EBD/OH/DB/MT) to identify drug names for adalimumab and include a comprehensive definition of discontinuation (Supplemental Table 1). Our search terms included Adalimumab, brand name Humira, and biosimilars brand names for article identification. Covidence was used for screening and abstraction [17].

Two independent reviewers screened each title and abstract, and completed full-text review, conflicts were resolved by a third reviewer. One reviewer completed data abstraction and a second reviewer validated the data. Nine reviewers were involved (EBD/JF/KB/YIG/DH/TL/CR/AS/MT/OH). Abstracted data included title, authors, publishing year, country, study design, number of centers, study duration, disease focus, subjects (N), deprescribed medications, discontinuation details, well-controlled disease definition, background medications, and limitations. Evidence levels were determined using the Oxford Center for Evidence-Based Medicine Levels of Evidence tool [18].

Descriptive statistics were completed, and key outcomes were compiled as reported by article authors or calculated including flare rate, recapture approach, and percentages. The results were stratified by disease population: RA, spondyloarthropathy, and JIA.

## Results

**Fig. 1:**
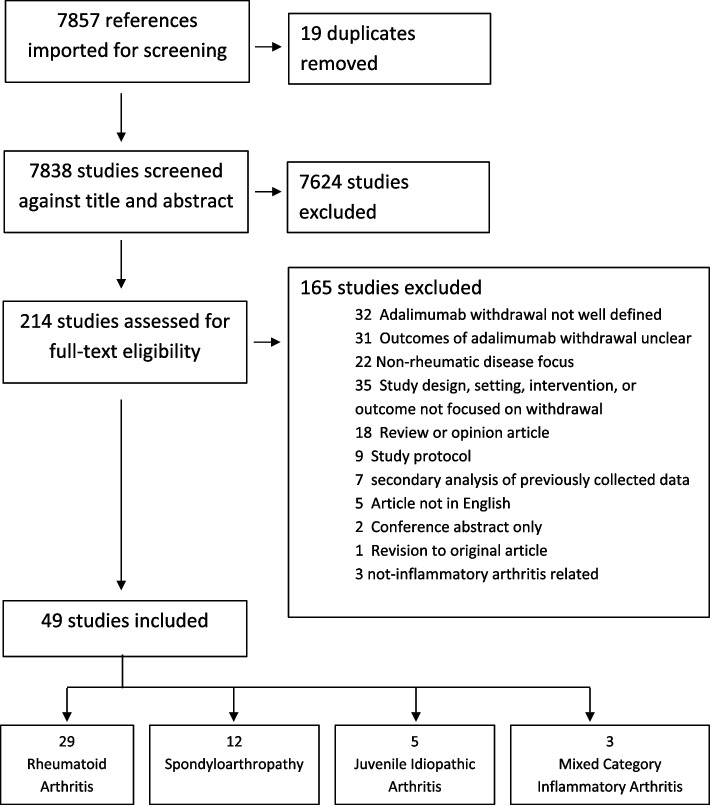
PRISMA diagram of manuscript selection. *Non-rheumatic disease defined as disease not primarily managed by a rheumatologist including GI conditions such as IBD and dermatologic conditions such as psoriasis

The search retrieved 7,838 studies, 214 were selected for full-text review, 49 for data abstraction (Fig. 1) including 12 randomized controlled trials (RCT), and 37 observational studies. Studies spanned 18 countries, 1 to 161 centers, with RCTs averaging 34 centers. In total, 5,682 patients were evaluated, and 3,657 attempted bDMARD discontinuation, with 1,418 treated specifically with adalimumab.

### Rheumatoid arthritis

Of 32 studies on RA, eight were RCTs (Table 1), of which 4 focused on adalimumab exclusively. Studies varied considerably in eligibility criteria, discontinuation approaches, and outcomes.

**Table 1:** Adult rheumatoid arthritis randomized controlled trial literature evaluating Adalimumab discontinuation, flare rates, recapture method and success rates

**First Author**	**Tascilar**	**VanMulligen**	**Emery**	**Bouman**
**Year**	2021	2020	2020	2017
**Study Design**	RCT	RCT	RCT	RCT
**Trial Name**	(RETRO)	(TARA 2Y)	(PREDICTRA)	(DRESS 3Y)
**Sites**	14	12	54	2
**Enrolled (N)**	299	189	122	180
**ADA specific taper**^**a**^	24 (13%)	40 (42%)	122 100%	42 (35%)
**Tapering method**	Dose reduction and abrupt stop	Dose reduction and prolonged dosing interval	Abrupt stop and prolonged dosing interval	Prolonged dosing interval
**Population RF/CCP prevalence**	RF 57% CCP 56%	RF 58% CCP 68%	NA	RF 80% CCP 73%
**Study Duration (months)**	12	24	9	36
**CID/LDA definition**	DAS28-ESR < 2.6 for 6 months	DAS ≤ 2.4 and SJC ≤ 1 at 2 time points within 3 months	DAS28-ESR/CRP < 2.6 for ≥ 6 months and DAS28-ESR < 2.6 at screening	LDA at 2 visits, determined by rheumatologist and DAS28-CRP
**Patient reported assessments**	PtGA, HAQ	HAQ-DI, SF-36, EQ-5D	HAQ-DI, PtGA, Rapid-3, SF-36, FACIT-fatigue	HAQ-QI, EQ5D
**Background therapy**	NA	100% csDMARDs in TNFi taper group	80% MTX	50% MTX
**Flare Rates**	Taper:43% (40/93)	TNFi 1st: 62%	Taper:36% (37/102)	Taper:
	Abrupt stop:55% (53/96)	csDMARD 1st: 61%	Abrupt stop:45% (9/20)	Major flare:17% (20/115)
	Continue:16/93 (17%)	(*p* = 0.84)		Minor flare:83% (96/115)
	No ADA specific data			Continue:14% (8/57)
**Recapture method**	Restart previous medication	Restart previous medication	ADA standard dosing	Restart previous medication
**Recapture results**	Taper: 62% (21/34)	46% at wk 12	38% (15/37) by wk 16	NA
	Abrupt stop: 76% (35/46)	67% at wk 24	Taper: 38% (11/29)	
			Abrupt stop: 50% (4/8)	
**Factors associated with successful discontinuation**	Autoantibody negativity, male gender, shorter disease duration, lower baseline DAS28	NA	Deep remission prior to medication discontinuation	NA
**Evidence quality**	1	1	1	1
**First Author**	**El Miedany**	**Fautrel**	**Chatzidionysiou**	**Smolen**
**Year**	2016	2016	2016	2014
**Study Design**	RCT	RCT	RCT	RCT
**Trial Name**	NA	(STRASS)	(ADMIRE)	(OPTIMA)
**Sites**	1	23	8	161
**Enrolled (N)**	157	138	31	207
**ADA specific taper**^**a**^	46 (37%)	29 (45%)	15 (100%)	102 (100%)
**Tapering method**	Abrupt stop and prolonged dosing interval	Prolonged dosing interval	Abrupt stop	Abrupt stop
**Population RF/CCP prevalence**	RF 50% CCP 60%	RF 68% CCP 80%	RF 58% CCP 66%	RF 95% CCP 90%
**Study Duration (months)**	12	18	12	12
**CID/LDA definition**	DAS28 < 2.6-ESR for 6 months, 3 visits	DAS28 < 2.6 for ≥ 6 months	DAS < 2.6 for 3 months at baseline and one more occasion 3–6 months prior	Stable LDA: DAS28-CRP < 3.2 at 22 or 26 weeks of treatment
**Patient reported assessments**	PROMS questionnaire	HAQ score	HAQ score	PtGA, Patient pain VAS, HAQ-DI
**Background therapy**	79% MTX	75% MTX or leflunomide	100% MTX	100% MTX
	21% other csDMARDs			
**Flare Rates**	Overall: 62% (77/125)	Taper:77% (49/64)	Abrupt stop:87% (13/15)	Abrupt stop: -DAS28 > 2.6: 33% (34/101) -DAS28 > 3.2: 18% (19/101)
	Tapered: 51% (32/63)	Continue:47% (34/74)	Continue:19% (3/16)	Continue: -DAS28 > 2.6: 14% (15/105) -DAS28 > 3.2: 8.5% (9/105)
	Abrupt stop: 73% (45/62)	(*p* = 0.0004)		
	Continue: 6.5% (2/32)			
**Recapture method**	Restart previous medication	Restart previous medication	ADA standard dosing	ADA standard dosing
**Recapture results**	100% (77/77) by week 16	41% (20/49) CID	90% (8/9) wk 12	NA
		39% (19/49) LDA	100% (9/9) wk 52^a^	
		8% (4/49) Moderate DA		
**Factors associated with successful discontinuation**	Lower DAS-28 score and better remission quality, Anti-CCP negativity, improving functional disability over 12 weeks, lower ultrasound greyscale and power doppler scores at 12 weeks	Spacing strategy, baseline HAQ score, RF negativity	Shorter disease duration, earlier ADA treatment start,, lower baseline DAS28 score	NA
**Evidence quality**	1	2	2	1

After data extraction, a study by Bouman et al. replaced Van Herwaarden et al., as both studies were from the DRESS trial, and Bouman reported longer follow up time

*RCT* randomized controlled trial, *ADA* Adalimumab, *CCP* cyclic citrullinated protein, *RF* Rheumatoid Factor, *TNFi* TNF inhibitor, *MTX* methotrexate, *csDMARD* conventional synthetic disease modifying antirheumatic drug, *VAS* visual analog scale, *PtGA* patient global assessment, *NA* not available, *Wk* week, *CID* clinically inactive disease, *LDA* low disease activity, *DA* disease activity, *SF-36* short form-36, *SJC* swollen joint count, *EQ5D* European Quality of Life-5 Dimensions, *HAQ-DI* Health Assessment Questionnaire

Disability Index. *CRP* C-reactive protein, *ESR* erythrocyte sedimentation rate

Recapture results reflect reported information only

Evidence quality was determined using the Oxford Centre for Evidence-Based Medicine: levels of Evidence (2009) guidance

^a^ADA specific indicates the number of patients who specifically tapered adalimumab

All RCTs required adalimumab dosing every other week before discontinuation, and 7/8 studies reported 50–100% concomitant csDMARD use. The minimum remission duration before RCT entry was variable (3–12 months, typically ≥ 6 months). Typically, disease flare was defined by disease activity score 28 (DAS28) ≥ 2.6 or delta-DAS28 ≥ 1.2, and well-controlled disease was defined as DAS28 < 2.6 in 16 studies and < 3.2 in 6 studies. The remainder used variable definitions for well-controlled disease. RA Boolean criteria for inactive disease [19] were used in three RA RCTs (Table 1). Patient reported outcomes, definition of clinically inactive disease (CID) and factors associated with successful medication discontinuation are reported in Table 1.

The flare rates after adalimumab discontinuation was variable between the studies depending on the population, discontinuation approach, and concomitant therapies (Table 1).

The 2020 PREDICTRA RCT [20] demonstrated higher flare rates by week 40 with abrupt stop of adalimumab, 45% (9/20) versus 36% (37/102) with tapering. Additionally, there was no association between adalimumab drug levels and the ability to maintain remission after tapering or abrupt stopping. The 2014 OPTIMA trial [21], which evaluated patients with early RA who achieved controlled disease for 12 months then withdrew adalimumab abruptly, reported flare rate (DAS28 > 2.6) with abrupt stop of 33% (34/101), compared to the continuation arm of 14% (15/105). LDA (DAS28 < 3.2) was maintained in 82% (82/101) of the adalimumab withdrawal arm versus 91% (96/105) in the continuation arm.

The 2016 ADMIRE RCT [22] reported differences in flare occurrence after discontinuation in patients in remission (DAS28 < 2.6) for at least 3 months, with 87% (13/15) flaring after abrupt stop versus 19% (3/16) of continuation subjects flaring, by 52 weeks. Notably, this trial had low enrollment, with only 33 of 237 eligible patients (14%) agreeing to participate, and shorter duration of inactive disease on medications prior to enrollment.

With respect to the sequence of discontinuation, the 2-year results of the 2019 TARA RCT [23] reported similar rates of flares between csDMARD-first versus TNFi-first, 61% versus 62% at 24 months (*p* = 0.84), but showed tendency to more complications with TNFi-first withdrawal. In the first year of the study, more patients who tapered TNFi-first lost Boolean remission (37% to 18%) versus those who tapered csDMARD-first (33% to 20%). Notably, TNFi-first participants were more likely to acquire erosions (12% versus 6%), and drug-free remission at 24-months was lower (11% versus 20%).

Twenty RA-specific non-RCTs were also reviewed. These studies demonstrated a large range of reported flares with abrupt stopping 5–75% and tapering 24–85%. Variability in the results reflected heterogeneity in study designs, populations, follow-up duration, and participants numbers (Supplemental Table 1). Considering long-term maintenance of disease control after discontinuation, the prospective cohort study in 2017, HOPEFUL-3 [24], evaluated adalimumab discontinuation in early RA disease. The authors reported that 3 years after adalimumab discontinuation, ~ 80% of patients maintained LDA versus 95% in the adalimumab + methotrexate continuation group, and that a lower DAS28-CRP cutoff value of 1.4 versus 2.0 at 2 years predicted LDA at 3 years.

### Spondyloarthropathy

Twelve studies examined outcomes after TNFi discontinuation in patients with spondyloarthropathies, eight evaluating spondyloarthritis (two RCTs), two psoriatic arthritis (one RCT), and two evaluating both (one RCT). Outcome data specific to adalimumab was scarce. Half of studies reported flare rates following TNFi discontinuation as a group (6/12, including all 4 RCTs), and when available, recapture data was often reported for the TNFi group (7/9, of which only 1 was an RCT). A majority of studies used the bath ankylosing spondylitis disease activity index (BASDAI) ≤ 2 to define inactive disease (*n* = 4) and LDA < 4 (*n* = 7) [25]. In studies using the ankylosing spondylitis disease activity score (ASDAS) [25] inactive disease was defined as < 1.3 (*n* = 2) and LDA < 2.1 (*n* = 2). In all instances, patients were in LDA or inactive disease for a minimum of 6 months before discontinuation. Patient reported outcomes, definition of CID, and factors associated with successful medication discontinuation are reported in Table 2.

**Table 2:** Adult Spondyloarthropathy literature evaluating Adalimumab withdrawal, flare rates, recapture method and success rates

**First Author**	**Michielsens**	**Wetterslev**	**Kwon**	**Gratacós**	**Landewé**	**Fong**
**Year**	2022	2022	2021	2019	2018	2016
**Study Design**	RCT non inferiority	Prospective cohort-registry	Retrospective cohort	RCT	RCT	Retrospective cohort
**Sites**	1	NA	1	22	107	1
**Enrolled (N)**	122	107	101	120	305	208
**ADA specific taper**^**b**^	62 (76%)	62 (58%)	101 (100%)	23 (40%)	140 (100%)	104 (50%)
**Tapering method**	Prolonged dosing interval	Dose reduction	Average dose quotients	Prolonged dosing interval	Abrupt stop	Prolonged dosing interval
**Study Duration (months)**	12	24	50	12	10	30
**Lowest ADA dose**	No ADA	No ADA	NA	40 mg Q3 weeks	no ADA	40 mg Q3 weeks
**CID/LDA definition**	PsA LDA: PASDAS ≤ 3.2 & mBSA ≤ 3% AS LDA: ASDAS < 2.1	BASDAI < 40; PGA VAS < 40; physician stated remission at time of enrollment; low disease activity on DANBIO (Danish registry) review for the prior year	BASDAI ≤ 4	BASDAI ≤ 2 and no clinically active arthritis or enthesitis and no CRP equal to or higher than the upper limit of normality for ≥ 6 months	ASDAS inactive disease score (< 1.3) at weeks 16, 20, 24, and 28 during open-label lead-in period	BASDAI < 4 for 56 months and not taking NSAIDs regularly
**Patient reported assessments**	PASDAS, ASDAS	BASDAI, BASFI, HAQ, HAQ-S, EQ-5D, ASAS HI, patient VAS for pain/fatigue/global	BASDI	ASDAS-CRP, BASDAI, VAS nocturnal axial pain	HAQ-S	BASDAI, DAS28-ESR
**Background therapy**	csDMARDS maintained, primarily MTX, leflunomide, NSAID	NA	Mixed, some on csDMARDs and NSAIDs	NA	csDMARDs, NSAIDs, steroids	Mixed; some csDMARDs
**Flare Rates**	Taper:69% (56/81) Continue:73% (30/41) (*p* = 0.32)	Taper: 100% (106/107) 27% (29) 2/3 dose 19% (21) 1/2 dose 27% (29) 1/3 dose 26% (28) stop	Taper: 44.6% (45/101)	Taper: 21.8% (13/58) Continue: 16.3% (8/55)	Stop: 53% (81/153) Continue: 29% (42/144)	Taper: 42% (20/48) AS: 42.4% (14/33) PSA: 40% (6/15)
**Recapture method**	Return to last effective dose of TNFi	4 week NSAID or steroid trial, if no response then return to last effective dose	ADA standard dosing	NA	ADA standard dosing	Restart previous medication
**Recapture results**	78%(18/23)	97% (104/107)	91.1% (41/45)	NA	57% (37/65) at 12 weeks	100% (48/48)^**a**^
**Factors associated with successful discontinuation**	none	Physician global VAS < 40 before medication change	Longer duration in LDA (> 5.3 months), higher average dose quotient (> 60%)	BASFI, lower hs-CRP	Lower ASDAS (0–1.3)	Lower disease activity in PsA patients
**Evidence quality**	1	2	2	1	1	2
**First Author**	C. Plasencia	A. Moverley	S. Arends	M. Almirall	H. Haibel	F. Cantini
**Year**	2015	2015	2015	2015	2013	2012
**Study Design**	Retrospective cohort	RCT	Prospective cohort	Prospective cohort	Secondary analysis	Case control
**Sites**	2	2	2	1	2	1
**Enrolled (N)**	117	17	58	42	46	131
**ADA specific taper**^**b**^	17 (23%)	5 (45%)	9 (16%)	27 (64%)	24 (100%)	53 (100%)
**Tapering method**	Prolonged dosing interval	Prolonged dosing interval	Prolonged dosing interval	Prolonged dosing interval	Prolonged dosing interval	Prolonged dosing interval
**Study Duration (months)**	12	3	24	12	24	36
**Lowest ADA dose**	40 mg Q6 weeks	no ADA	40 mg Q4 weeks	40 mg Q3 weeks	no ADA	40 mg Q4 weeks
**CID/LDA definition**	LDA or remission DAS28 < 3.2 for 6 months prior to taper	LDA: MDA criteria, stable disease for proceeding 6 months as indicated by treating physician	LDA: BASDAI < 4, ASDAS < 2.1 and CRP < 5 mg/L CID: ASDAS < 1.3	LDA: BASDAI between 2 and 4 units, allowing peripheral disease and increased CRP levels	LDA: ASAS40	CID: psoriatic: 6 M of no fatigue or pain, no symptoms, normal CRP/ ESR psoriatic spondylitis:6 M of BASDAI ≤ 4, no joint symptoms, normal CRP/ESR. No NSAIDs or corticosteroids for 2 visits
**Patient reported assessments**	NA	VAS, SF36, HAQ	ASQoL	NA	ASDAS, SF-36 (physical, mental component), BASFI, pain scores	NA
**Background therapy**	No background therapy for ADA; for overall group, some on MTX	Mixed; some MTX	Mixed; some NSAIDs and csDMARDs	Mixed; some csDMARDs	NA	Mixed; some were taking MTX
**Flare Rates**	Taper: 30% (22/74) ADA: 11.8% (2/17)	Taper: 54.6% (6/11) Continue: 0% (0/6)	Overall: 47% (27/58) ADA: 44% (4/9)	Overall: 23.8% (10/42) ADA: 22.2% (6/27)	ADA: 79% (19/24)	ADA: 11.3% (6/53)
**Recapture method**	Restart previous medication	NA	Restart previous medication	NA	ADA standard dosing	ADA standard dosing
**Recapture results**	100% (2/2)	NA	most—(n) not reported	NA	63% (12/19) at 1 yr 73% (15/20) at 2 yr	100% (6/6)
**Factors associated with successful discontinuation**	Male sex	NA	Lower ASQoL	Longer disease duration, biologic treatment duration, and remission duration	NA	NA
**Evidence quality**	2	2	2	2	1	3

*RCT* Randomized controlled trial, *ADA* Adalimumab, *NA* not available, *yr* year, *AS* ankylosing spondylitis, *PSA* psoriatic arthritis, *LDA* low disease activity, *csDMARD* conventional synthetic disease modifying anti-rheumatic drug, *CRP* C-reactive protein, *ESR* erythrocyte sedimentation rate, *HAQ-DI* Health Assessment Questionnaire, *SF-36* short form-36, *VAS* visual analog scale, *ASDAS* Axial Spondyloarthritis Disease Activity Score, *BASFI* Bath Ankylosing Spondylitis Functional Index, *ASQoL* Bath Ankylosing Spondylitis Functional Index, *DAS28* disease activity score 28, *ASAS-HI* assessment of spondyloarthritis health index, *PASDAS* Psoriatic Arthritis Disease Activity Score, *EQ5D* European Quality of Life-5 Dimensions, *NSAID* non-steroidal anti-inflammatory drug, *mBSA* modified body surface area

Recapture results reflect reported information only

^a^recapture was achieved in LDA

^b^ADA specific indicates the number of patients who specifically tapered adalimumab

Evidence quality was determined using the Oxford Centre for Evidence-Based Medicine: levels of Evidence (2009) guidance

Most medications were tapered by prolonged dosing interval (9/12, of which 3 were RCTs). Dose was decreased in one study, and prolonged intervals and decreased doses were combined in one study, while the 4th RCT abruptly stopped medication. A majority of studies allowed background therapy, commonly nonsteroidal anti-inflammatory drugs (NSAIDs) and csDMARDs (Table 2). The 4 RCTs noted flares in 22–85% of participants withdrawing TNFi, although only Landewé et al*. *[26] reported adalimumab-specific data, with a flare rate of 53% (81/153) following abrupt stop. Flare rates among non-RCTs ranged from 11–100%, although a majority of studies reported flares between 20–47% (Table 2). Notably, in the study with a 100% flare rate, patients were eligible to continue tapering medications until complete discontinuation unless a flare occurred [27], while the study with the lowest flare rate maintained adalimumab use every 4 weeks [28].

In the treat-to-target dose reduction RCT by Michielsens et al*.* [29]*,* 56/81 (69%) patients maintained LDA in the TNFi taper arm (77% adalimumab) compared with 73% (30/41, with 28/41 on adalimumab), who continued medications, demonstrating noninferiority with a mean reduction in the overall TNFi medication dose of 47% in the taper arm. At 12 months, 72% remained tapered, of whom 28% were able to stop TNFi completely.

Another prospective, pragmatic TNFi discontinuation trial from Denmark [27] reported less favorable outcomes, where 33% of patients were able to taper adalimumab, but only 1% in the overall TNFi cohort stopped medication successfully at 2-years. Notably, 50% of flares occurred when the medication dose was reduced more than 50%. A lower physician global assessment (PhGA) score was a predictor of successful tapering, with the suggestion of improved outcomes with lower baseline MRI erosion scores.

The RCT by Gratacós et al*.* [30] showed a similar risk of flares at one year regardless of disease status; with 23% flares in patients who tapered medication after achieving clinical remission, vs 20% for those who tapered in LDA.

### Juvenile idiopathic arthritis

Five observational pediatric studies addressed adalimumab discontinuation (Table 3). Six months of clinically inactive disease (CID) using ACR preliminary criteria [31, 32] was required prior to discontinuation in all but 1 study [33], which used ACR/EULAR remission criteria [19]. Background therapies varied between the studies. Most studies reported outcomes among TNFi as a single class. Flare rates for abrupt TNFi stopping ranged widely (28–82%), with flares typically occurring within 7 months. The largest cohort [4] reported a 37% (39/105) overall TNFi flare rate with a 28.5% (4/14) adalimumab specific flare rate within 10 months of discontinuation. Patient reported outcomes, definition of CID and factors associated with successful medication discontinuation are reported in Table 3.

**Table 3:** Pediatric literature evaluating Adalimumab withdrawal, flare rates, recapture method and success rates

**First Author Year**	**S. Papailiou 2021**	**C. Liao 2021**	**D. Lovell 2018**	**C. Chang 2015**	**E. Iglesias 2014**
**Study Design Sites**	Retrospective cohort 2	Retrospective cohort 1	Prospective cohort 16	Retrospective cohort 1	Retrospective cohort 1
**Enrolled (N)** **ADA specific taper**^**a**^	35 35 (100%)	75 14 (27%)	137 14 (13%)	335 25 (18.5)	18 1 (5%)
**Tapering method**	Prolonged interval and decreased dose	Prolonged dosing interval	Abrupt Stop	Abrupt stop	Abrupt stop
**Study Duration (months)**	24	Minimum 6 Median 6 years	8–10	Mean: 33 (2–95)	6
**Disease focus**	35 ERA (100%)	75 ERA (100%)	18 Oligo (13.1%) 17 RF + Poly (12.4%) 102 RF- poly (74.5%)	239 poly (71%) 96 ERA (29%)	9 RF—poly (50%) 5 Oligo (28%) 3 Undifferentiated (17%) 1 ERA (5%)
**CID/LDA definition**	2011 ACR/EULAR remission criteria and JSpADA index for 6 M	Wallace Criteria for 6 M	Wallace Criteria for 6 M	Wallace Criteria for 6 M	Wallace Criteria for 6 M
**Patient reported assessments**	NA	NA	PtGA, CHAQ	NA	NA
**Background therapies**	NA	csDMARDs tapered prior to TNFi taper	background therapies maintained	Mixed, some patients tapered MTX first, some maintained MTX	csDMARDS discontinued prior to TNFi withdrawal
**Flare Rates**	Taper: 17% (6/35) 3/35 (8.5%) 1 episode of monoarthritis treated with steroid injection	Taper: 63% (33/52) 50% of flares within 12 M	Taper: 37% (39/106) ADA: 28.5% (4/14)	Taper: 42% (31/73) at 6 M 63% (39/62) at 12 M TNFi 1st: 47% 6 M, 78% 12 M MTX 1st: 16% 6 M, 19% 12 M	Taper: 82% (14/17)
			Mean time to flare: 7.07 M (SEM 0.3)	Mean time to flare: 7.2 M ± 9.1	Mean time to flare: 3 M (SD 2)
**Recapture method**	ADA standard dosing	NA	NA	Restart previous medication	Restart previous medication
**Recapture results**	5/6 (83%) restarted ADA 1/6 (17%) steroid therapy or biologic	NA	NA	45/71 (63%)	14/14 (100%)
**Factors associated with successful discontinuation**	Rapid initiation of ADA in disease course	Shorter disease duration prior to first CID (trend towards significance)	Shorter disease duration prior to first CID	Duration of TNFi therapy < 12 M at time of withdrawal	csDMARD withdrawal prior to TNFi withdrawal
**Evidence quality**	2	2	2	5	2

*ERA* enthesitis related arthritis, *ADA* Adalimumab, *CID* clinically inactive disease, *TNFi* TNF inhibitor, *MTX* methotrexate, *csDMARD* conventional synthetic disease modifying antirheumatic drug, *NA* not available, *SD* standard deviation, *SEM* standard error measurement, *Poly* Polyarticular, *Oligo* Oligoarticular, *RF* Rheumatoid Factor, *JSPADA* Juvenile Spondyloarthritis Disease Activity

^a^ADA specific indicates the number of patients who specifically tapered adalimumab

Evidence quality was determined using the Oxford Centre for Evidence-Based Medicine: levels of Evidence (2009) guidance

Flare rates for studies evaluating TNFi taper were only reported in patients with enthesitis related arthritis (ERA) and were variable, ranging from 17% (6/35) for patients followed for 24 months after taper initiation [32] to 63% (33/52) for patients followed for a median of 6 years after discontinuation [33]. No pediatric studies focused on maintaining remission with the lowest possible dose (Table 3).

The two adalimumab taper studies used different approaches. Papailiou et al*.* [33] reported that 40% who tapered adalimumab underwent a 50% dose reduction, and the remaining 60% spaced medication intervals to 3 weeks, with both groups stopping medication at 3 months. Conversely, Liao et al*.* [34] allowed for a clinician-led tapering scheme with a consensus of increasing dosing intervals during tapering.

#### Recapture after disease flare

In all three disease categories when flare occurred the withdrawn medication was commonly restarted at standard dosing, (14 RCTs), although some trials returned to the last effective dose (1 RCT). Despite this consistent approach, recapture success after flare was variable among disease groups.

### Rheumatoid arthritis

For studies with recapture information (18/29), recapture rates were high. In non-RCT studies (37–100%) and in RCTs (80–100%), although definition of recapture often included LDA. Time to recapture also varied. El Miedany [35] reported 100% recapture in all patients regardless of the tapering approach. In studies comparing discontinuation approaches, recapture rates were higher after an abrupt stop versus gradual taper (50% vs. 38% and 76% vs. 62%, respectively) [20, 36]. The ADMIRE RCT [22] reported 100% recapture of participants by week 52 after abrupt stop. Conversely, the PREDICTRA RCT reached only 50% recapture by week 16 [20].

### Spondyloarthropathy

Only two spondyloarthropathy RCTs reported recapture data: Landewé et al*.* [26] reported 57% (37/65) recapture by 12 weeks after an abrupt stop, and Michielsens et al*.* [29] incorporated recapture into their treat-to-target strategy and reported 78% (18/23) of patients re-achieved LDA when returned to full dose, and 17% (5/23) required biologic switching. The prospective cohort by Weterslev et al*.* [27] reported 75% (104/107) recapture after taper, with time to recapture not reported.. In the observational studies with recapture data, 73–100% of patients were recaptured.

### Juvenile idiopathic arthritis

Three of the five observational pediatric studies included recapture data. Chang et al*.* [37] reported recapture of CID in 63% (45/71) of patients with ERA. Smaller studies demonstrated higher rates in subjects with multiple subtypes of JIA up to 100% (14/14) [38], and 83% (5/6) [31] in patients with ERA (Table 3). The pediatric data, although limited, suggests that recapture rates may be similar between tapering (63–100%) and abrupt stopping (83%).

## Discussion

Multiple adult and a few pediatric studies have evaluated adalimumab discontinuation in patients with well-controlled inflammatory arthritis, though most studies focused on TNFi as a whole instead of reporting adalimumab specific outcomes. Studies demonstrated differences in populations, demographic and clinical characteristics, disease duration at discontinuation, use of background therapies, and varied discontinuation approaches and outcome measures. As a whole, the available literature suggests that adalimumab taper is possible and often trialed, with certain cohorts having comparable outcomes with either continued or tapered medication. Reported flare rates vary widely, with higher flare rates in patients who abruptly stop medication compared to those who taper. Additionally disease recapture may be more likely after abrupt stop than medication taper.

Based on evaluation of the studies with the highest level of evidence (large RCTs), available only in those with RA and spondyloarthropathy, our conclusions corroborate recent reviews and guidelines focused on tapering practices for bDMARDs, indicating that tapering should be considered after maintaining disease control [15, 39–42].

Considering the criteria for inactive and controlled disease, the variable criteria for study inclusion made comparison of outcomes difficult within and across populations. Most RA and spondyloarthropathy studies allowed for LDA, while JIA studies mostly required strict CID for at least 6 months [32]. Consequently, it may prove more difficult for patients with JIA to remain “flare free” by CID criteria, whereas RA or spondyloarthropathies could be in LDA without reporting a flare. This discrepancy in flare incidence may impact the recapture incidence, as an increased flares creates greater opportunity to observe recapture outcomes.

Similar to the stringent CID criteria for remission in JIA, the RA Boolean criteria for inactive disease [19] were used in three RA RCTs in this review. When used, Boolean criteria demonstrated a decrease in patients who sustained remission with taper [43]. Wetterslev et al*.* [44] also reported a lower baseline PhGA score was associated with successful dose reduction of TNFi in subjects with spondyloarthropathy. Overall, this may indicate that the use of stringent remission definitions or less tolerance of LDA may improve outcomes after medication discontinuation.

There was heterogeneity in discontinuation approach strategies even within disease specific RCTs. Several RA RCTs compared abrupt stop and tapering in the same study, where two earliest RCTs evaluated abrupt stopping without a taper arm [21, 22]. Conversely, the spondyloarthropathy studies, focused on dosing intervals and lowest possible dose to maintain disease control. The earliest pediatric studies [4, 37, 38] focused on the abrupt stop, while tapering was more prevalent in recent studies. While heterogeneous, these findings may indicate a trend toward tapering towards lowest effective dose and away from an abrupt stop.

Specific tapering approaches also varied. The most common approach was gradual spacing of adalimumab in single week intervals to complete discontinuation, guided by patient disease activity. Although some used 50% dose reduction and spacing to every four weeks as the initial step. In three RA RCTs, dosing interval was increased several times prior to stopping [20, 45, 46], one increased the interval first with subsequent dose reduction [47], and two decreased TNFi dose without changing the interval [35, 36]. Within spondyloarthropathy and pediatric studies, increased interval was the predominant approach.

Considering outcomes after treatment discontinuation, to date, no specific predictors for subpopulations with the highest success rates for adalimumab discontinuation have been identified. Several studies in this review noted Boolean remission, early disease control (duration, chronicity, erosions, time from disease onset to start of TNF therapy) and low initial PhGA as possible predictors. Since most studies evaluated adult RA patients and RF factor positivity did not stand out as a risk factor. This is likely different for the JIA population in whom RF positivity is associated with severe disease [48]. Some studies noted higher flare rates with TNFi discontinuation prior to csDMARD discontinuation, as reported in the JIA [37] and the RA population [49]. Notably, variation in flare rates may be attributable to variable follow-up duration, the shortest being 3 months in spondyloarthritis [50] and 6 months in JIA, while all RA studies followed patients for 9 or more months.

Adalimumab-specific findings for dose dependence are also similar to other TNF trials, noting that a 50% taper is possible for most patients, while complete discontinuation often leads to flares. Two retrospective studies in axial spondyloarthritis [51, 52] point to a higher flare risk when tapering beyond 25–50%, where approximately 50% of patients were able to decrease the medication dose with comparable rates of LDA in the taper and continuation arms. These findings are similar to those reported in studies evaluating certolizumab (C-OPTIMISE trial) [53] and golimumab (GO-BACK trial) [54] dose reduction in spondyloarthritis and supports tapering medication to maintain LDA target.

The importance of recapture after flare, to both clinicians and the patients, cannot be understated. The most common approach was restart of the standard drug dose and interval. Less often, a return to the previous effective dose was used [27, 29, 45, 46]. Recapture rates were high in all populations, however we call to attention the lower recapture rates after taper versus abrupt stop in all three populations. Some investigators incorporated recapture data into overall outcomes, noting that taper is reasonable, even in the face of flare, in the setting of a high expectation of disease recapture. This approach is different from the available pediatric csDMARD data, where treatment escalation is often needed after flare [55]*.* These findings suggest that the recapture of disease control is often possible and probable.

A number of gaps in the literature were noted. Although several RCTs have focused on RA, fewer RCTs focused on spondyloarthropathy, and none on JIA. Only 12/25 studies reported adalimumab outcome data separately from other TNFis. Length of follow-up after medication discontinuation was variable and is problematic, as short durations may miss delayed flares and result in an overestimation of drug-free remission. Heterogeneity approaches created difficulty in comparisons across studies.

Furthermore, patient-reported outcomes (PROs) were not consistently reported across studies [20] although an example of utilization of these measures is seen in the TARA and DRESS trials [23, 45]. Incorporating PROs and shared decision making regarding discontinuation strategy into study design should be highly considered, as they are critical for accurate disease monitoring, patient satisfaction, and may improve enrollment and retention. Furthermore, as patients and families often cite increased worry surrounding side effects and the financial and psychological burden of medications as the reason for discontinuation [12], we propose systematic PRO collection when conducting medication discontinuation trials.

Limitations of this review include possible erroneous exclusion of articles, and exclusion of abstracts as these may add novel trial information. This review was limited by reporting of adalimumab specific data, and limited pediatric data. Further we did not focus on drug or anti-drug antibody levels during the discontinuation process, or the pediatric uveitis population, two avenues for future research.

## Conclusion

The current literature suggests that outcomes are generally favorable for adalimumab tapering in inflammatory arthritis patients with well-controlled disease. However, the probability of flares ranges broadly, from 17–100%, with limited data in JIA and spondyloarthropathy. Recapture rates with standard dosing of adalimumab are high, up to 80–100%, however, recapture may be higher after abrupt stopping than tapering. Discontinuation approach and outcomes reported are variable across studies and disease types. Further investigation of adalimumab specific discontinuation approaches will be useful for shared decision making surrounding medication discontinuation.

## Supplementary Information

Supplementary Material 1.

## Data Availability

Data sharing is not applicable to this article as no datasets were generated or analyzed during the current study.

## Abbreviations

TNFI: Tumor necrosis factor inhibitors
JIA: Juvenile idiopathic arthritis
RA: Rheumatoid arthritis
RCT: Randomized controlled trial
bDMARD: Biologic disease-modifying antirheumatic drugs
csDMARD: Conventional synthetic DMARDs
LDA: Low disease activity
ACR: American College of Rheumatology
EULAR: European Alliance of Associations for Rheumatology
DAS28: Disease activity score 28
CID: Clinically inactive disease
BASDAI: Bath ankylosing spondylitis disease activity index
ASDAS: Ankylosing spondylitis disease activity score
NSAID: Non-steroidal anti-inflammatory drug
PhGA: Physician global assessment
ERA: Enthesitis related arthritis
PRO: Patient-reported outcomes
