# Supplementary material for: Approaches and outcomes of adalimumab discontinuation in patients with well-controlled inflammatory arthritis: a systematic search and review
Source: Pediatr Rheumatol Online J. 2024 Dec 30;22:112. doi: 10.1186/s12969-024-01046-3 (PMC11684048; doi:10.1186/s12969-024-01046-3)
Supplement: Supplementary file 1 — Supplementary Material 1. [file 12969_2024_1046_MOESM1_ESM.docx]

| **Supplementary Table S1: Keywords and indexing terms for the search** | |
| --- | --- |
| PubMed | (Adalimumab[mesh] OR Adalimumab OR Humira OR Amjevita OR Cyltezo OR "antibody d2e7" OR Adaly OR amgevita OR amsparity OR cinnora OR exemptia OR hadlima OR hefiya OR halimatoz OR hulio OR hyrimoz OR idacio OR imraldi OR mabura OR solymbic OR trudexa) AND ("drug administration schedule"[mesh] OR "withholding treatment"[mesh] OR "drug administration schedule" OR withhold* OR withheld OR "exit strategy" OR "exit strategies" OR withdraw* OR tapering OR "dose reduction" OR discontinu* OR "dosage reduction" OR "dose decrease" OR "dosage decrease" OR "reduction of drug dose" OR wean* OR "decreasing frequency" OR "decreasing dose" OR "stopping medication" OR "drug survival") |
| EMBASE | ((Adalimumab/exp OR Adalimumab OR Humira OR Amjevita OR Cyltezo OR ‘antibody d2e7’ OR Adaly OR amgevita OR amsparity OR cinnora OR exemptia OR hadlima OR hefiya OR halimatoz OR hulio OR hyrimoz OR idacio OR imraldi OR mabura OR solymbic OR trudexa) AND (‘treatment withdrawal’/exp OR ‘drug dose reduction’/exp OR ‘drug administration schedule’ OR withhold* OR withheld OR ‘exit strategy’ OR ‘exit strategies’ OR withdraw* OR tapering OR ‘dose reduction’ OR discontinu* OR ‘dosage reduction’ OR ‘dose decrease’ OR ‘dosage decrease’ OR ‘reduction of drug dose’ OR wean* OR ‘decreasing frequency’ OR ‘decreasing dose’ OR ‘stopping medication’ OR ‘drug survival’)) NOT ‘conference abstract’:it |
| CINAHL | ((SU Adalimumab OR Adalimumab OR Humira OR Amjevita OR Cyltezo OR ‘antibody d2e7’ OR Adaly OR amgevita OR amsparity OR cinnora OR exemptia OR hadlima OR hefiya OR halimatoz OR hulio OR hyrimoz OR idacio OR imraldi OR mabura OR solymbic OR trudexa) AND (SU ‘drug admistration schedule’ OR SU ‘treatment duration’ OR SU ‘drug tapering’ OR ‘drug administration schedule’ OR withhold* OR withheld OR ‘exit strategy’ OR ‘exit strategies’ OR withdraw* OR tapering OR ‘dose reduction’ OR discontinu* OR ‘dosage reduction’ OR ‘dose decrease’ OR ‘dosage decrease’ OR ‘reduction of drug dose’ OR wean* OR ‘decreasing frequency’ OR ‘decreasing dose’ OR ‘stopping medication’ OR ‘drug survival’)) |
| Cochrane | (Adalimumab OR Humira OR Amjevita OR Cyltezo OR "antibody d2e7" OR Adaly OR amgevita OR amsparity OR cinnora OR exemptia OR hadlima OR hefiya OR halimatoz OR hulio OR hyrimoz OR idacio OR imraldi OR mabura OR solymbic OR trudexa) AND ("drug administration schedule" OR "withholding treatment" OR "drug administration schedule" OR withhold OR withholding OR withheld OR "exit strategy" OR "exit strategies" OR withdraw OR withdrawing OR withdrawal OR tapering OR "dose reduction" OR discontinue OR discontinued OR discontinuation OR discontinuing OR "dosage reduction" OR "dose decrease" OR "dosage decrease" OR "reduction of drug dose" OR wean OR weaning OR weaned OR "decreasing frequency" OR "decreasing dose" OR "stopping medication" OR "drug survival") |

| **Author** | **Year** | **Study Design** | **Disease Focus** | **Enrolled**  **(N)** | **ADA Specific taper***  **n (%)** | **Study Duration**  **(Months)** | **Tapering method** | **Flare rates** | **Recapture Rates** | **Evidence Quality** |
| --- | --- | --- | --- | --- | --- | --- | --- | --- | --- | --- |
| **M. Fornaro** | 2021 | Prospective cohort | Multiple: PsA, axSpA and RA | 85 | 22 (59) | 12 | Prolonged dosing interval | Overall: 40% (15/37) ADA: 14% (3/22) | NA | 4 |
| **C. Murphy** | 2015 | Prospective cohort | Multiple: RA, PsA, AnkSp | 79 | 34 (43) | 24 | Prolonged dosing interval | 44% (35/79) | NA | 2 |
| **J. Inciarte-Mundo** | 2013 | Retrospective cohort | Multiple: RA, AS, PsA, JIA, MCTD, SAPHO, Stills) | 169 | 19 (27) | 12 | Prolonged dosing interval | 11% (4/35) | NA | 2 |
| **M. Ochiai** | 2021 | Case control | RA | 43 | 4 (9) | 12 | Abrupt Stop | Overall: 42% (18/43) ADA: 25% (1/4) | NA | 3 |
| **T. Naniwa NA** | 2020 | Prospective cohort | RA | 135 | 15 (16) | 12 | Prolonged interval and decreased dose | Overall: 35% (33/95) ADA: 26% (4/15) | 88% (23/26) | 2 |
| **A Yamaguchi** | 2020 | Prospective cohort | RA | 52 | 52 (100) | 60 | Abrupt Stop | 74% (31/46) | 71% (17/24) | 2 |
| **S. Ito** | 2019 | Prospective cohort | RA | 130 | 26 (100) | 24 | Abrupt Stop | 5% (1/20) | 100% (1/1) | 4 |
| **N. Kimura** | 2019 | Retrospective cohort | RA | 122 | 4 (100) | 20 | Abrupt Stop | 75% (3/4) | 67% (2/3) | 2 |
| **C. Brahe** | 2019 | Prospective cohort | RA | 143 | 24 (32) | 24 | Prolonged dosing interval | 85% (122/141) | 85% (102/122) | 2 |
| **O. Vittecoq** | 2019 | Prospective cohort | RA | 53 | 8 (15) | 18 | Prolonged dosing interval | Overall: 36% (19/53), ADA: 25% (2/8) - 6M taper phase Overall: 75% (38/51), ADA: 87% (7/8) - 12M discontinuation phase | NA | 2 |
| **F. Lamers-Karnebeek** | 2018 | Secondary analysis | RA | 210 | 210 (100) | 12 | Abrupt Stop | 51% (106/210) within 12M | NA | 1 |
| **Y. Tanaka** | 2017 | Prospective cohort | RA | 172 | 74 (100) | 48 | Abrupt Stop | 20% (15/74) | NA | 2 |
| **F. Ibrahim** | 2017 | Prospective cohort | RA | 103 | 54 (55) | 12 | Prolonged dosing interval | Overall: 0-6M: 19% (9/47), 7-12m: 27% (11/40) ADA: 33% (18/54) | NA | 2 |
| **C. Bouman** | 2017 | Prospective cohort | RA | 42 | 42 (25) | 36 | Prolonged dosing interval | 38% (16/42) Able to dose reduce: 36% (15/42) | NA | 2 |
| **M. Cárdenas** | 2017 | Prospective cohort | RA | 10 | 10 (15) | 24 | Prolonged dosing interval | 41% (28/68) within 12M  59% (40/68) within 24M ADA: 40% (4/10) within 12M, 60% (6/10) within 24M | 90% (36/40) | 2 |
| **Y. Tanaka** | 2016 | Prospective cohort | RA | 220 | 96 (100) | 6.5 | Abrupt Stop | 15% (14/92) within 12M 50% (38/96) within 24M | NA | 2 |
| **C. Plasencia** | 2016 | Retrospective cohort | RA | 144 | 23 (34) | 12 | Prolonged dosing interval | Overall: 39% (26/67) ADA: 39% (9/23) | NA | 2 |
| **S. Alivernini** | 2016 | Prospective cohort | RA | 42 | 22 (52) | 6 | Prolonged dosing interval | Overall: 30.9% (13/42) ADA: 33% (7/21) | 100% (13/13) | 4 |
| **D. Chen** | 2015 | Prospective cohort | RA | 64 | 64 (100) | 6 | Decreased dose | 23.5% (15/64) within 6M | NA | 2 |
| **S. Hirata** | 2013 | Prospective cohort | RA | 197 | 50 (100) | 12 | Abrupt Stop | 41% (21/51) within 6M | 83% (5/6) | 2 |
| **Y. Tanaka** | 2013 | Prospective cohort | RA | 75 | 52 (100) | 39 | Abrupt Stop | 40% (21/52) | 100% (21/21) | 2 |
| **M. Harigai** | 2012 | Retrospective cohort | RA | 46 | 22 (100) | 12 | Abrupt Stop | 36% (8/22) | 37% (3/8) | 4 |
| **O. Brocq** | 2009 | Prospective cohort | RA | 28 | 5 (24) | 12 | Abrupt Stop | 75% (15/20) within 12M | 100% (15/15) | 4 |
| **A. Den Broeder** | 2002 | Prospective cohort | RA | 21 | 21 (100) | 12 | Decreased dose | 85% (18/21) | 94% (17/18) | 2 |
| Footnotes:  *ADA specific indicates the number of patients who specifically tapered adalimumab RCT: randomized controlled trial. ADA: Adalimumab. CCP: cyclic citrullinated protein. RF: Rheumatoid Factor.TNFi: TNF inhibitor. MTX: methotrexate. csDMARD: conventional synthetic disease modifying antirheumatic drug. NA: not available. Wk: week. CID: clinically inactive disease. LDA: low disease activity. DA: disease activity Recapture results reflect reported information only  Evidence quality was determined using the Oxford Centre for Evidence-Based Medicine: levels of Evidence (2009) guidance | | | | | | | | | | |
